# Compositional Assessment of Honeys from the Central Atlantic Forest: Multielement and Physicochemical Characterization

**DOI:** 10.1021/acsomega.5c06565

**Published:** 2025-10-12

**Authors:** Letícia Rosário Silveira, Jaílson Santos de Novais, Caio Silva Assis Felix, Gabriela Pereira Costa, Guilherme Piloto Santos, Allison Gonçalves Silva, Mário Marques Silva Junior

**Affiliations:** † Instituto de Humanidades, Artes e Ciências, 423878 Universidade Federal do Sul da Bahia, Campus Sosígenes Costa, Porto Seguro, Bahia 45810-000, Brazil; ‡ Centro Interdisciplinar de Energia & Ambiente, CIEnAm, 28111 Universidade Federal da Bahia, Salvador, Bahia 40170-115, Brazil; § 169704 Instituto Federal de Educação, Ciência e Tecnologia da Bahia, Campus Porto Seguro, Porto Seguro, Bahia 45810-000, Brazil

## Abstract

The ongoing rise in both consumption and exportation of Brazilian honey underscores the need for stricter authentication and traceability protocols to ensure the quality and safety of this food product. Therefore, this study characterizes the multielemental and physicochemical composition of honey samples from the Central Corridor of the Atlantic Forest—a recognized yet threatened biodiversity hotspot in Brazil. Using inductively coupled plasma optical emission spectrometry and standard physical-chemical methods, 23 honey samples were analyzed for mineral content, including Al (0.0013–0.109 mg kg^–1^), Ca (28.9–262.49 mg kg^–1^), Cr (0.09–1.36 mg kg^–1^), Cu (0.051–0.467 mg kg^–1^), Fe (0.8–26.3 mg kg^–1^), K (83.8–1896.5 mg kg^–1^), Mg (20.8–349.5 mg kg^–1^), Mn (0.010–6.94 mg kg^–1^), Na (10.51–464.16 mg kg^–1^), Se (0.143–0.317 mg kg^–1^), Sr (0.003–1.429 mg kg^–1^), and Zn (0.142–4.276 mg kg^–1^), and quality parameters such as moisture (11.69–19.84%), insoluble solids (0.050–0.571 g 100 g^–1^), acidity (41.3–79.5 mequiv kg^–1^), HMF (7.80–39.77 mg kg^–1^), diastase activity (3.07–23.76 DN), reducing sugars (56.31–80.07 g 100 g^–1^), and apparent saccharose (2.10–17.74 g 100 g^–1^). Potassium, calcium, and magnesium were the predominant minerals. Principal component analysis differentiated honey types, with polyfloral and monofloral samples forming distinct groups. Most samples met the Brazilian and international standards for physicochemical parameters. The findings highlight the quality profile of the Atlantic Forest’s honey, which may contribute to the conservation of this endangered biome.

## Graphical abstract

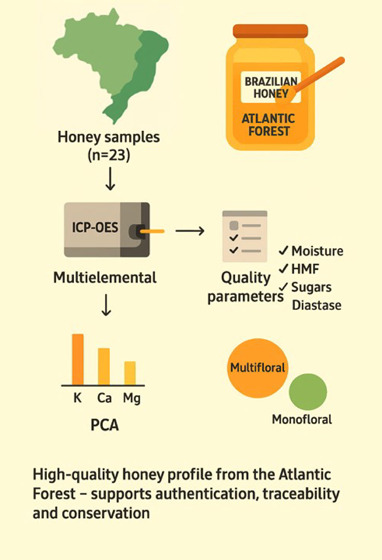

## Introduction

In an increasingly globalized society, ultraprocessed foods have become a quick and easy source of nourishment. Healthy, natural food is becoming harder to find yet increasingly necessary. Healthy eating is fundamental to the promotion of well-being, as it provides quality nutrients that are essential for the body to function properly, which also helps to prevent diseases.^1,2^ As a natural food, honey has a wide range of nutritional and medicinal properties. It is rich in sugars, vitamins, minerals, proteins, and antioxidants,^3^ so it can be a healthy substitute for refined sugar. In addition, its antimicrobial and anti-inflammatory properties make it valuable for both nutrition and traditional therapeutic practices.^4^ Incorporating honey into a balanced diet can help promote a healthier life, with benefits to both physical health and emotional well-being, providing a naturally sweet and nutritious flavor.^5,6^

Honey is a product of primarily botanical origin, produced by honey bees from the nectar of flowers, secretions from living parts of plants, and secretions from plant-sucking insects. These raw materials are processed by metabolic enzymes and stored in honeycomb in the hives.^7–9^ Honey is mostly composed of glucose, fructose, and water, making it a saturated sugar solution. In trace amounts, honey also contains proteins, free amino acids, and pollen grains along with additional minor constituents that contribute to its characteristic organoleptic properties. These attributes are influenced by botanical and geographical origin, soil composition, seasonal variations, as well as harvesting, processing, and storage practices.^10–13^ Even in small proportions, these substances act as a “fingerprint” and can be used to identify the honey’s origin and establish quality criteria. Sweeteners or other substances that modify the honey’s composition cannot be added, as they would make the product adulterated or tampered.^14,15^

Varieties of chemical compounds are found in honey. Because of this, their composition often undergoes changes during storage. These changes can influence both its nutritional and sensory properties, often associated with chemical reactions, such as the Maillard reaction, which is triggered by heat.^16,17^ In addition, due to commercial factors, honey can be stored for long periods, up to almost a year, before being consumed. The flavors and colors resulting from this process can be considered desirable or undesirable,^18^ depending on the context. These transformations can occur slowly throughout the storage time or more quickly upon being subjected to high temperatures.

In the marketing of honey, the practice of adding sugars or other substances that alter the original composition is considered a fraud. The criteria employed in honey marketing and regulatory evaluation encompass: (a) sensory properties—including color, flavor, aroma, and texture; (b) maturity indices—such as reducing sugars, moisture, and apparent sucrose; (c) purity parameters—notably the presence of pollen grains, water-insoluble matter, and minerals; and (d) deterioration markers—including fermentation, acidity, diastase activity, and hydroxymethylfurfural levels.^18–20^ Honey can also be classified by the type of processing it undergoes, drained, pressed, or centrifuged, in order to remove insect parts, pollen grains, and wax particles. Numerous studies have been carried out in Brazil and around the world to evaluate and classify the honey produced in different regions, identifying various physical and chemical parameters.^21–23^

Minerals (Cu, Fe, Mn, Zn, etc.) are present in honey at low percentages, ranging from 0.04% to 0.2% due to geographical and seasonal influences. Others, such as Ca, K, Na, and Mg, can have increased values due to environmental conditions.^19,24–26^ Evaluating the mineral composition of foods with high sugar content can generate spectral interferences during quantification, due to the need for rigorous pretreatment of the sample to eliminate undesirable substances that could alter the analysis. Various analytical methods can be used to study the composition of honey, most notably atomic spectrometry, or more specifically, inductively coupled plasma optical emission spectrometry (ICP-OES), an analytical technique that is widely used to determine metal content due to its multielement analysis, sensitivity, versatility, and robustness.

The characterization of honey for the assessment of its quality, authenticity, and traceability, given the botanical and geographical variability of the samples, generates a significant amount of data.^27,28^ Given the complexity, the application of principal component analysis (PCA) becomes an effective chemometric tool. PCA reduces the dimensionality of the data, identifying correlation patterns between elements and revealing natural groupings of samples, often related to geographical origin, botany, or production practices.^29,30^ The integration of data obtained by ICP-OES analysis with PCA constitutes a robust and efficient methodology for honey analysis, providing information about its composition, authenticity, and food safety. This can be positive in view of the quality parameters for controlled designation of origin (DOC), allowing samples from different regions and blooms to be distinguished, consolidating this strategy as an advanced tool for quality control and traceability in the beekeeping industry.^31–33^

This study aims to evaluate the multielement and physicochemical parameters of honey produced and sold in an area of the Central Atlantic Forest Corridor in southern Bahia, using ICP-OES. This region of tropical forest is internationally recognized for its rich plant biodiversity. As one of the world’s biodiversity hotspots, the Atlantic Forest is home to a wide variety of endemic plant species, which directly influence the chemical and nutritional composition of honey. With the increasing suppression of vegetation due to strong anthropic pressure, with high rates of deforestation and fragmentation, the evaluation of local products such as honey can act as a bioindicator of environmental conditions, providing data on possible impacts related to contamination.^34,35^ The physical-chemical and multielement evaluation and characterization of this product not only allows for verification of its authenticity and quality but also for the valuation of the ecosystem services provided by bees. Additionally, beekeeping has become an important source of income for local producers, who benefit from the richness of the flora as a sustainable form of income generation. Furthermore, this study presents a chemometric application using PCA to evaluate and classify honey samples based on the parameters indicated above.

## Materials and Methods

### Materials and Reagents

All reagents used were analytical grade. All solutions used were prepared using ultrapure water (18.2 MΩ cm^–1^) supplied by a Master System MS3000 (Gehaka, São Paulo, Brazil). All glassware used was previously decontaminated by immersion in a 10% (v/v) HNO_3_ solution for 24 h and rinsed with ultrapure water before use. Nitric acid 65% (m/m) and hydrogen peroxide 30% (m/m), both from Merck (Darmstadt, Germany), were used to prepare the analysis curves and to digest the honey samples. Standard calibration solutions (Al, Ca, Cr, Fe, K, Mg, Mn, Na, Se, Sr, and Zn) were prepared for the appropriate dilution of the stock reference solutions at concentrations of 1000 or 4000 mg L^–1^ (Titrisol, Merck, Germany).

The reagents used in physicochemical characterization were all of analytical grade and prepared according to established protocols for each method. Free, lactonic, and total acidities were determined by titration with sodium hydroxide (Merck, Germany) and hydrochloric acid (Merck, Germany). For the quantification of hydroxymethylfurfural (HMF), the samples were treated with Carrez I (potassium ferrocyanide, Sigma-Aldrich, USA) and Carrez II (zinc acetate, Sigma-Aldrich, USA) solutions and sodium bisulfite (Merck, Germany). For the determination of reducing sugars and apparent sucrose, sucrose was used as a standard (Sigma-Aldrich, USA), along with Fehling’s solutions modified by Soxhlet (copper sulfate and double sodium and potassium tartrate, Sigma-Aldrich, USA), hydrochloric acid, and sodium hydroxide. The determination of water-insoluble solids was performed by gravimetry using phloroglucinol (Sigma-Aldrich, USA) and sulfuric acid (Merck, Germany). The diastase activity of the samples was evaluated by enzymatic reactions with sodium acetate and acetic acid (Merck, Germany), anhydrous soluble starch (Sigma-Aldrich, USA), and iodine and potassium iodide (Sigma-Aldrich/Merck), in addition to sodium chloride (Merck, Germany). Lund, Fiehe, and Lugol reactions were conducted using tannic acid, ether (Merck, Germany), resorcinol (Sigma-Aldrich, USA), and iodine and potassium iodide (Sigma-Aldrich/Merck). All solutions were prepared with ultrapure water.

### Physicochemical Analysis

The physicochemical characterization was conducted to assess key honey quality parameters, including: (a) maturity, determined by reducing sugars, moisture content, and apparent sucrose; (b) purity, evaluated through water-insoluble solids; and (c) deterioration, indicated by total, free, and lactone acidity, diastase activity, and hydroxymethylfurfural concentration. These parameters were analyzed using *Physicochemical Methods for Food Analysis*—*CAP*. *VII*—*Sugars and Related Products*, from the Adolfo Lutz Institute.^36^ All analyses were carried out in triplicate.

The moisture index was obtained by drying the samples in an oven (Lucadema, LUCA-80/100) until the mass no longer showed any significant variation. To determine insoluble solids, the honey samples were solubilized in ultrapure water at 80 °C and vacuum filtered. To check if there was still sugar in the filtered material, drops of sulfuric acid and an alcoholic solution of phloroglucinol were added. To determine the amount of reducing sugars, the inverted sugar (glucose + fructose) content was calculated using the modified Lane and Eynon method and, for apparent sucrose, the sugars, after inversion by acid hydrolysis, were determined using the modified Lane and Eynon method.^28^

Acidity (total, free, and lactone) was determined through neutralization volumetry. The honey samples were titrated with 0.05 mol L^–1^ sodium hydroxide and 1% (m V^–1^) phenolphthalein alcohol solution as an indicator to determine free acidity. Lactone acidity was titrated with 0.05 mol L^–1^ of hydrochloric acid through the addition of excess sodium hydroxide. The total acidity of the samples was calculated by adding the free and lactone acidity. The pH of the samples was also measured using a pH meter (Mettler Toledo, FIVE EASY F20).^28^

Diastase activity was determined by using a UV/vis spectrophotometer (YOKE-UV1200PRO) at a wavelength of 660 nm. For this procedure, solutions of starch, iodine, acetate buffer (pH 5.3), and 0.5 mol L^–1^ sodium chloride were prepared. The starch solution was standardized with a 0.00035 mol L^–1^ iodine solution. During this stage, 10 g of honey was dissolved in water and buffered with acetate solution before the addition of sodium chloride. After preparation, the solution was mixed with starch and placed in a water bath at 40 °C. Aliquots were then taken every 5 min and mixed with iodine solution to measure the absorbance.^28^

The HMF content was determined using UV/vis spectrophotometry at wavelengths of 284 and 336 nm. Solutions of Carrez I and II and a solution of 0.2% m V^–1^ sodium bisulfite were prepared. A honey sample of 5 g was weighed, dissolved in water, and transferred to a volumetric flask into which Carrez solutions were added for clarification. The sample was then filtered, and two 5 mL aliquots were taken, one diluted in water (sample) and the other in sodium bisulfite (reference). Both samples were subjected to an ultrasonic bath (model SSDC20L-110, SOLIDSTEEL—São Paulo, Brazil) at a frequency of 40 kHz and power of 160 W, maintained at 25 °C for 3 min prior to measurement.^28^

### Multielement Analysis through ICP-OES

#### Instrumentation

The multielement analysis of the honey samples was carried out with an ICP-OES (iCAP PRO X, Thermo Fisher Scientific, Massachusetts, USA), with axial visualization. A solid-state detector was used for the simultaneous determination of the analytes of interest (Al, Ca, Cr, Fe, K, Mg, Mn, Na, Se, Sr, and Zn). The optical system was calibrated with a stock solution of various elements for ICP-OES, while a standard solution of 5.0 mg L^–1^ of Mn was used for optical alignment. Spectral lines were selected according to the absence of spectral interference and adequate sensitivity to determine elements at high and low concentrations according to the emission lines of the elements under investigation. Lines that exhibited low interference, high analytical signal, and high background ratios were selected. The ICP-OES parameters were radio frequency power generator wattage (1250 W), plasma gas rate (13 L min^–1^), auxiliary gas rate (0.5 mL min^–1^), nebulizer gas rate (0.5 mL min^–1^), and injector tube diameter (2.5 mm). The analytical wavelengths (nm) used for multielement analysis were Al (309.271), Ca (396.366), Cr (283.563), Fe (259.940), K (766.490), Mg (279.553), Mn (257.610), Na (588.995), Se (196.090), Sr (407.771), and Zn (202.548).

#### Sample Preparation for Multielemental Analysis by ICP-OES

Twenty-three honey samples of *Apis mellifera* L., 1758 (*Apidae*: *Apini*), produced and marketed by local beekeepers in municipalities of southern Bahia, northeast Brazil, were acquired. The samples were identified and coded (Table 1) according to the predominant bloom indicated by the producer and kept in their original containers in the dark at room temperature. The classification of monofloral and multifloral honeys was based solely on producer information, and no melissopalynological analysis was performed.

**Table 1:** Honey Samples from *A. mellifera* Bees Collected in the Central Corridor of the Atlantic Forest, Brazil

code	honey floral source
SAF1	multifloral honey
EAL	eucalyptus honey
EAV	eucalyptus honey
LMERC	orange blossom honey
SAP	eucalyptus honey
SAB	eucalyptus honey
SAJ	multifloral honey
SAL	multifloral honey
SPM	multifloral honey
SAV	multifloral honey
SBM	multifloral honey
SFB	multifloral honey
SG	multifloral honey
SAJ1	multifloral honey
SAJ 2	multifloral honey
SAV 1	multifloral honey
SAV 2	multifloral honey
SPM 1	multifloral honey
SPM 2	multifloral honey
SBM1	multifloral honey
EAF	eucalyptus honey
SAF	multifloral honey
VF	*velame* honey
SAF2	multifloral honey

The honey samples were submitted to a digestion process using a Milestone Ethos Easy microwave oven (Sorisole (BG)—Italy) with an infrared temperature sensor and a capacity of 24 PTFE vials. For this purpose, samples of approximately 0.5 g were weighed into polytetrafluoroethylene (PTFE) tubes with 5.0 mL of HNO_3_ and 1.0 mL of H_2_O_2_. The digestion procedure was carried out as described in Table 2.

**Table 2:** Microwave-Assisted Digestion Protocol for Honey Samples

time (min)	power (W)	temperature (°C)
00:10:00	1800	160
00:15:00	1800	210
00:10:00	1800	210

Upon completion of the procedure, the obtained solutions were transferred to precleaned and decontaminated tubes and subsequently stored under controlled refrigeration conditions (−5 °C) until the quantification step.

## Results and Discussion

### Analytical Merit Parameters

The physicochemical analyses carried out in this study followed the methodology presented by the Adolf Lutz Institute.^29^ The analytical technique used to determine the chemical elements in honey samples is called ICP-OES. The limits of detection (LOD), quantification (LOQ), and precision (expressed as the relative standard deviation) for determining metal composition are described in Table S1.

Due to the absence of a certified reference material similar to the honey sample matrix because of its complexity, the method achieved precision using the analyte addition technique.^37^ The results obtained and expressed as a 95% confidence interval were Al 98–102%, Ca 91.0–107.6%, Cr 99.0–107.0%, Fe 101.0–117.0%, K 94.0–109.0%, Mg 86.0–103.0%, Mn 100.0–108%, Na 98.0–116%, Se 85.0–104%, Sr 85.0–100.0%, and Zn 100.0–110.0%. The results showed satisfactory recovery at a 95% confidence level.

Honey is a natural food of great nutritional importance, being an excellent source of quick energy due to its high concentrations of fructose and glucose, which are easily absorbed sugars. It is also rich in antioxidants, such as phenolic compounds and flavonoids, which help fight free radicals and reduce oxidative stress, helping to prevent chronic diseases.^38^ Honey also contains essential minerals, such as potassium, calcium, and magnesium, which contribute to proper metabolic functioning and boost the immune system. The mineral content found in the honey samples is shown in Table 3.

**Table 3:** Mineral Concentrations in *A. mellifera* Honey Samples Collected in the Central Corridor of the Atlantic Forest, Brazil^a^

sample	Al (mg kg^–1^)	Ca (mg kg^–1^)	Cu (mg kg^–1^)	Cr (mg kg^–1^)	Fe (mg kg^–1^)	K (mg kg^–1^)	Mg (mg kg^–1^)	Mn (mg kg^–1^)	Na (mg kg^–1^)	Se (mg kg^–1^)	Sr (mg kg^–1^)	Zn (mg kg^–1^)
EAF	>LOD	254.72 ± 2.18	0.11 ± 0.02	0.467 ± 0.019	8.36 ± 0.03	1975.0 ± 13.4	130.11 ± 0.5	6.94 ± 0.053	217.22 ± 1.67	>LOD	>LOQ	1.416 ± 0.015
EAL	>LOD	153.6 ± 0.64	>LOQ	0.062 ± 0.002	26.3 ± 1.5	894.6 ± 6.9	97.8 ± 0.4	4.09 ± 0.023	370.94 ± 1.712	0.244 ± 0.012	0.775 ± 0.007	4.276 ± 0.016
EAV	0.057 ± 0.001	192.7 ± 1.40	>LOD	>LOQ	7.6 ± 0.1	853.0 ± 1.1	268.5 ± 1.0	3.63 ± 0.038	64.17 ± 0.491	0.160 ± 0.044	1.429 ± 0.015	0.322 ± 0.005
LMERC	>LOD	215.4 ± 1.63	>LOD	>LOQ	21.1 ± 0.0	1001.3 ± 7.8	143.8 ± 0.1	1.24 ± 0.005	118.80 ± 0.696	0.143 ± 0.026	0.634 ± 0.001	0.747 ± 0.004
SAB	>LOQ	85.8 ± 0.99	>LOQ	>LOD	17.5 ± 0.2	766.7 ± 6.9	51.7 ± 0.4	1.69 ± 0.016	52.18 ± 0.578	0.231 ± 0.032	0.275 ± 0.004	0.634 ± 0.011
SAF1	0.109 ± 0.106	189.0 ± 1.73	>LOD	>LOD	10.4 ± 0.1	83.8 ± 2.4	40.8 ± 0.4	0.07 ± 0.002	21.86 ± 0.285	0.200 ± 0.036	0.645 ± 0.010	0.241 ± 0.005
SAF2	>LOD	92.37 ± 0.47	0.11 ± 0.02	0.085 ± 0.006	6.55 ± 0.04	923.97 ± 15.59	35.83 ± 0.14	2.88 ± 0.008	31.91 ± 0.04	>LOQ	>LOQ	0.907 ± 0.007
SAJ	>LOD	77.9 ± 0.85	>LOD	>LOD	1.3 ± 0.0	477.9 ± 6.7	79.2 ± 0.6	1.83 ± 0.017	90.18 ± 1.272	0.207 ± 0.027	0.093 ± 0.003	1.025 ± 0.008
SAJ 2	>LOD	73.46 ± 1.20	0.25 ± 0.79	0.058 ± 0.012	6.92 ± 0.01	745.81 ± 0.66	47.21 ± 0.21	1.35 ± 0.005	92.73 ± 1.07	>LOD	>LOQ	0.961 ± 0.004
SAJ1	>LOD	262.49 ± 2.92	1.31 ± 0.06	0.07 ± 0.00	9.10 ± 0.01	1896.5 ± 19.12	349.5 ± 3.8	3.06 ± 0.013	59.32 ± 0.41	>LOD	>LOD	2.017 ± 0.023
SAL	0.096 ± 0.003	42.9 ± 0.23	>LOQ	>LOD	0.8 ± 0.0	85.6 ± 1.7	40.4 ± 0.4	0.010 ± 0.003	10.51 ± 0.159	0.229 ± 0.024	>LOQ	0.372 ± 0.004
SAP	0.018 ± 0.002	94.9 ± 1.40	>LOQ	>LOD	9.0 ± 0.1	290.7 ± 7.2	41.1 ± 0.4	0.84 ± 0.013	47.65 ± 0.864	0.162 ± 0.028	1.083 ± 0.009	2.265 ± 0.036
SAV	>LOD	228.2 ± 1.10	>LOQ	>LOD	1.3 ± 0.0	162.7 ± 2.3	173.4 ± 1.7	2.18 ± 0.028	295.00 ± 3.229	0.211 ± 0.031	0.919 ± 0.012	0.335 ± 0.009
SAV 1	>LOQ	100.35 ± 0.54	0.25 ± 0.93	0.051 ± 0.011	7.85 ± 0.02	1257.37 ± 12.18	58.61 ± 0.55	1.02 ± 0.020	43.41 ± 0.43	>LOD	>LOQ	0.612 ± 0.011
SAV 2	>LOQ	110.07 ± 0.76	1.36 ± 0.08	0.059 ± 0.019	8.29 ± 0.01	1299.95 ± 12.57	61.25 ± 0.42	1.05 ± 0.002	84.65 ± 0.68	>LOQ	>LOQ	0.661 ± 0.007
SBM	>LOQ	37.8 ± 0.46	>LOD	0.218 ± 0.004	10.7 ± 0.1	135.1 ± 2.2	23.6 ± 0.3	0.23 ± 0.005	13.83 ± 0.10	0.251 ± 0.020	0.003 ± 0.002	0.490 ± 0.013
SBM1	>LOD	101.29 ± 0.78	0.15 ± 0.03	0.085 ± 0.022	6.8 ± 0.01	892 ± 4.48	35.00 ± 0.03	2.79 ± 0.014	37.79 ± 0.03	>LOD	>LOD	2.075 ± 0.017
SFB	>LOD	28.9 ± 0.31	>LOD	>LOD	9.1 ± 0.1	547.4 ± 8.6	20.8 ± 0.1	0.13 ± 0.003	15.28 ± 0.206	0.213 ± 0.017	>LOD	0.142 ± 0.001
SG	0.013 ± 0.001	194.9 ± 0.19	>LOD	>LOD	3.7 ± 0.1	140.64 ± 0.9	140.6 ± 0.9	0.71 ± 0.007	134.67 ± 1.273	0.317 ± 0.068	0.0792 ± 0.010	0.997 ± 0.014
SPM	0.054 ± 0.001	137.1 ± 1.62	>LOQ	>LOD	38.3 ± 0.4	1110.8 ± 9.3	52.6 ± 0.5	0.33 ± 0.005	10.56 ± 0.078	0.222 ± 0.042	0.151 ± 0.005	2.775 ± 0.027
SPM 1	>LOQ	87.97 ± 0.30	0.73 ± 0.08	0.056 ± 0.001	6.23 ± 0.02	768.68 ± 6.31	50.76 ± 0.17	1.45 ± 0.002	106.54 ± 0.24	>LOQ	>LOQ	1.210 ± 0.006
SPM 2	>LOQ	143.5 ± 1.73	0.09 ± 0.41	0.231 ± 0.005	7.99 ± 0.02	1119.2 ± 10.82	59.92 ± 0.44	1.23 ± 0.007	96.88 ± 0.88	>LOD	>LOD	3.834 ± 0.017
VF	>LOD	166.87 ± 0.31	0.09 ± 0.03	0.1390 ± 0.016	9.52 ± 0.40	633.17 ± 7.52	42.24 ± 0.14	0.56 ± 0.004	464.16 ± 2.32	>LOQ	>LOD	0.767 ± 0.007
CODEX ALIMENTARIUS	*	*	*	*	*	*	*	*	*	*	*	*
MERCOSUL (GMC 89/99)	*	*	*	*	*	*	*	*	*	*	*	*

a *Not specified.

The concentrations of metals were significantly heterogeneous due to the diversity of the Brazilian flora, which contains more than 36,600 species of angiosperms.^39^ The vast majority of samples are multifloral and are termed by their producers as wild honey. In Brazil, most honey is of wild origin, as reflected by the pollen spectrum of samples studied in different regions across the country.^40^ Even samples labeled as monofloral, while showing a predominant floral source, reveal a polyfloral palynological profile.^41^

Potassium was found to have the highest values, ranging from 83.8 mg kg^–1^ (SAF2 multifloral honey) to 1896.5 mg kg^–1^, with the SAJ1 (multifloral honey) sample having the highest potassium content. The wide variation in potassium content among honey samples is primarily attributed to the botanical origin of the nectar. This is particularly relevant in samples classified by producers as wild honey, with no specific floral source, as well as to the mineral composition of the soil in the collection sites, which differs across the extensive study area. In addition, the presence of pollen grains and the limited degree of filtration of the final product are contributing factors that account for the elevated concentration of this macroelement in certain honey types.

This was followed by magnesium (20.8–349.53 mg kg^–1^), calcium (28.92–228.02 mg kg^–1^), and sodium (21.86–464.16 mg kg^–1^). These levels are in line with values found in the literature, which show that these metals are predominant in honeys.^15,30,42,43^ The lowest values found in honey were for aluminum (0.013–0.109 mg kg^–1^), copper (0.09–1.31 mg kg^–1^), iron (1.3–21.3 mg kg^–1^), manganese (0.01–4.09 mg kg^–1^), selenium (0.143–0.317 mg kg^–1^), and zinc (0.241–4.27 mg kg^–1^).

Potassium and magnesium are among the minerals found in high concentrations in honey, representing the largest fractions of its mineral composition, regardless of its botanical or geographical origin. This may be directly related both to the composition of the nectar of the plants visited by bees and to the physiological processes of *A. mellifera* itself.^44–47^ The high concentration of potassium can be explained by its natural abundance in plant tissues and floral nectars, where it plays key roles in osmoregulation and cellular metabolism. Similarly, magnesium is present at high levels due to its central role as an enzymatic cofactor and structural component of chlorophyll, directly reflecting the botanical origin of the nectar.^48,49^

The aluminum (0.013–0.109 mg kg^–1^) and chromium (0.051–0.467 mg kg^–1^) levels found in the honey samples analyzed are generally below the limits suggested by international regulatory agencies, including Codex Alimentarius (FAO/WHO), the Brazilian Health Regulatory Agency (ANVISA, RDC 722/2022), and the European Food Safety Authority. However, the presence of these metals, even at relatively low levels, highlights the need for continuous monitoring due to the potential for adverse effects on human health, especially with cumulative exposure. These results reinforce the importance of detailed assessment of composition and implementation of strict controls in honey production, ensuring its safety and authenticity in accordance with international food safety standards.

### Physicochemical Parameters of Honey

The physical and chemical parameter values showed that the honey samples differed considerably in terms of their constitutions (Table 4). The honeys analyzed had significant acidity values, some with values above the established value of 50 mEq/kg.^8^ This may be due to the fact that southern Bahia is an important producer of citrus fruits, giving this characteristic to its honey.^50,51^ In addition, the SAV honey sample had a higher apparent reducing sugar content than the other samples, which can be attributed to the higher fraction of monosaccharides, mainly glucose and fructose.

**Table 4:** Measured Values of Physical and Chemical Parameters in *A. mellifera* Honey Samples Collected from the Central Corridor of the Atlantic Forest, Brazil

sample	moisture content (%)	insoluble solid content (g 100 g^–1^)	total acidity (meq kg^–1^)	diastase activity (DN)	HMF (mg kg^–1^)	reducing sugar (g 100 g^–1^)	apparent saccharose (g 100 g^–1^)
SAF1	17.63 ± 1.09	0.069 ± 0.002	48.5 ± 1.07	15.34 ± 0.09	34.9 ± 0.04	66.53 ± 0.01	7.18 ± 0.04
EAL	11.69 ± 1.23	0.056 ± 0.021	43.5 ± 1.83	20.61 ± 1.03	26.49 ± 0.01	60.75 ± 0.19	2.62 ± 1.01
EAV	17.43 ± 2.38	0.053 ± 0.003	48.7 ± 1.13	14.41 ± 1.09	17.36 ± 0.12	63.65 ± 0.07	12.31 ± 0.02
LMERC	14.82 ± 0.64	0.064 ± 0.001	63.1 ± 0.02	3.10 ± 1.30	9.6 ± 2.04	60.81 ± 0.03	2.1 ± 0.04
SAP	16.05 ± 1.93	0.052 ± 0.014	72.7 ± 0.96	13.56 ± 0.46	32.39 ± 0.01	67.83 ± 1.39	17.74 ± 1.38
SAB	18.76 ± 1.25	0.051 ± 0.001	55 ± 0.74	13.68 ± 0.05	32.3 ± 0.08	69.89 ± 1.06	6.89 ± 1.64
SAJ	16.44 ± 2.37	0.052 ± 0.017	53 ± 1.01	18.76 ± 0.98	19.34 ± 0.12	65.14 ± 0.08	3.72 ± 0.28
SAL	15.11 ± 2.41	0.051 ± 0.006	64 ± 0.37	10.64 ± 0.00	39.77 ± 0.02	56.31 ± 0.6	2.51 ± 0.72
SPM	14.82 ± 1.03	0.051 ± 0.082	59.7 ± 1.05	10.19 ± 0.20	20.5 ± 1.06	64.59 ± 0.23	2.00 ± 0.76
SAV	16.73 ± 1.72	0.054 ± 0.002	79.5 ± 1.06	23.76 ± 0.49	31.88 ± 1.01	80.07 ± 1.05	11.47 ± 0.93
SBM	15.09 ± 2.09	0.050 ± 0.106	41.3 ± 0.08	19.93 ± 0.27	25.44 ± 0.02	63.19 ± 0.07	6.9 ± 0.02
SFB	19.84 ± 0.16	0.059 ± 0.081	43.5 ± 0.06	6.64 ± 0.07	23.24 ± 0.93	66.56 ± 1.08	7.54 ± 1.05
SG	17.64 ± 1.46	0.058 ± 0.007	43.55 ± 1.85	3.07 ± 1.05	7.8 ± 2.04	65.98 ± 0.01	8.41 ± 0.67
SF	13.73 ± 2.68	0.054 ± 0.058	58 ± 0.10	18.83 ± 0.65	13.74 ± 0.02	66.42 ± 0.76	7.13 ± 0.82
SBE	15.69 ± 1.82	0.056 ± 0.533	53 ± 0.09	7.88 ± 0.36	11.28 ± 1.68	62.2 ± 1.74	3.84 ± 0.91
SAJ1	18.84 ± 0.58	0.0688 ± 0.011	48.4 ± 0.91	11.86 ± 1.29	19.86 ± 0.03	63.27 ± 0.04	5.64 ± 0.60
SAJ 2	17.66 ± 2.08	0.0599 ± 0.041	48.3 ± 0.09	16.76 ± 0.06	21.2 ± 1.72	66.71 ± 1.1	8.61 ± 0.07
SAV 1	15.97 ± 0.75	0.0582 ± 0.976	50.6 ± 0.60	17.00 ± 1.42	23.56 ± 0.05	63.16 ± 1.7	5.16 ± 0.02
SAV 2	16.65 ± 2.17	0.0578 ± 0.892	47.7 ± 0.94	6.85 ± 0.06	21.28 ± 1.06	65.42 ± 0.03	4.33 ± 0.21
SPBM 1	15.60 ± 1.08	0.543 ± 0.865	48.3 ± 0.06	9.18 ± 1.01	17.8 ± 0.02	60.77 ± 0.02	6.01 ± 0.11
EAF	16.55 ± 1.59	0.5137 ± 0.062	48.9 ± 0.11	14.06 ± 0.06	22.37 ± 0.04	58.22 ± 0.02	3.06 ± 0.23
SAF2	17.63 ± 1.58	0.571 ± 0.060	45.4 ± 0.90	11.26 ± 1.14	17.48 ± 0.02	66.7 ± 0.093	3.46 ± 0.10
VF	14.25 ± 2.01	0.485 ± 2.005	47.8 ± 0.30	13.17 ± 0.93	19.53 ± 1.01	65.9 ± 0.02	2.15 ± 0.18
CODEX ALIMENTARIUS^55^	20	0.1	80	8	40	60	5
MERCOSUL (GMC 89/99)^56^	20	0.1	50	8	60	65	6

The amount of moisture in honey depends directly on when it is harvested. The southern region of Bahia has a significant amount of rainfall throughout the year, associated with the degree of maturity reached by the hive. Assessing this parameter is important, as it is directly linked to the longevity of the honey. A relatively high moisture content causes instability in the honey over time and increases the chances of the product fermenting, spoiling, and losing its flavor, lowering the quality of the honey.^52^ The 23 samples had moisture values between 11.69 and 19.84%, showing an adequate degree of maturity when harvested. These values are in line with those defined in Brazilian legislation.^8^

The amount of insoluble solids can vary depending on the bloom related to the honey, other materials collected by the bees during foraging, and the composition of the hive, but in most cases, it is low. Of the 23 samples studied, 18 had values between 0.050 and 0.069%, below the permitted maximum value (0.1%). However, the VF, EF, SPM, and SF samples had values above this, which may be the result of handling the product during filtering to remove material such as pollen, wax, propolis, or fragments of the bees themselves.

HMF is an indicator of honey quality and freshness. As a byproduct generated from the degradation of the sugars contained in honey, it occurs naturally during storage and can be intensified through superheating.^53,54^ The HMF values found in the honey samples ranged from 7.80 to 39.77 mg kg^–1^. These values are within the limits set by national and international standards,^55,56^ which indicate a maximum of 60 mg kg^–1^. The variation may be associated with the use of smoke during honey harvesting, storage time (some samples are sold at street markets), or other physicochemical properties, such as moisture content and acidity.

Another important parameter is diastase activity, which corresponds to the enzyme activity contained in 1 g of honey, which in turn can hydrolyze 0.01 g of starch in 1 h at 40 °C.^57,58^ The values described in Brazilian legislation are 8 Göthe units, except for honeys with low natural enzymatic activity, such as citrus honeys, which must have at least 3 Göthe units as long as the HMF content does not exceed 15 mg kg^–1^. Several factors influence the enzymatic content of honey, such as the age of the bees, the period of nectar collection, the physiological period of the colony, and the sugar content.^34^ All honey samples evaluated were within the parameters stipulated by national legislation, with LMERC and VF featuring particularly as they showed activity close to 3 Göthe units and did not exceed the legal limit for HMF (15 mg kg^–1^).

The values for the amount of reducing sugars ranged from 56.31 to 69.89 g 100 g^–1^ of honey, which meets the requirements of the Ministry of Agriculture and Livestock^8^ (MAPA). The values found match those from the literature for honeys produced in Brazil. The sucrose content in honey samples ranged from 1.35 to 17.74 at 100 g^–1^. Of the 23 samples analyzed, 13 had values below the maximum permitted limit (6 g 100 g^–1^), while 10 exceeded this limit and were identified as outliers. The presence of these outliers may reflect different factors: (a) fraud, involving the addition of external sugars; (b) early harvesting, which prevents the complete enzymatic conversion of sucrose into glucose and fructose; or (c) specific characteristics of the botanical origin of the nectar, particularly from plants naturally rich in sucrose. For example, samples such as SAP (17.74 g 100 g^–1^) and EAV (12.31 g 100 g^–1^), with significantly high levels, may indicate premature harvesting or botanical influence, while others may represent deliberate adulteration, requiring complementary analyses, such as isotopic or chromatographic profiling, for confirmation. In contrast, samples with low sucrose levels are consistent with proper maturation processes and indicate high quality with no signs of adulteration. Detailed analysis of these outliers is crucial to differentiate natural variations from those resulting from illegitimate practices, allowing for a more accurate assessment of honey authenticity and quality.^59–61^

### Evaluation of Results Using the Multivariate Analysis Technique

#### Evaluation by Principal Component Analysis

The analysis of the 23 honey samples involved measuring the content of 11 metals (aluminum, calcium, chromium, iron, potassium, manganese, magnesium, sodium, selenium, strontium, and zinc) and physicochemical parameters (moisture content, insoluble solids content, total acidity, diastase activity, HMF, reducing sugar, and apparent sucrose). This resulted in a data matrix (23 × 18) with the metals and physicochemical parameters as columns and samples as rows. Because the values found for each analysis were of widely varying magnitudes, it was necessary to rescale them to keep them in the same proportion. The data obtained were then evaluated using the PCA technique.

Figure 1 shows the variability explained by the principal components, with the first four together retaining 65.62% of the total variance of the data. The load values of the corresponding variables are listed in Table 5. The first principal component (PC1), responsible for 24.64% of the variance, was strongly associated with potassium, chromium, selenium, and insoluble solids, all of which were positively correlated. This combination of variables suggests that PC1 is mainly related to differences in the mineral composition and purity of honey, reflecting variations of botanical and geographical origin. The second principal component (PC2), which explained 16.12% of the variance, was mainly determined by calcium, magnesium, and strontium, elements that may be directly linked to the edaphic characteristics of the collection region, functioning as potential geochemical markers. The third component (PC3), responsible for 12.19% of the variance, had moisture as the predominant variable, indicating its relevance in discriminating samples based on the degree of maturation and processing. Finally, the fourth principal component (PC4), which represented 10.63% of the variance, was mainly influenced by calcium, manganese, potassium, and diastase activity, suggesting the joint contribution of enzymatic and mineral factors in the additional differentiation of samples, not captured by the first three components.

**Figure 1:**
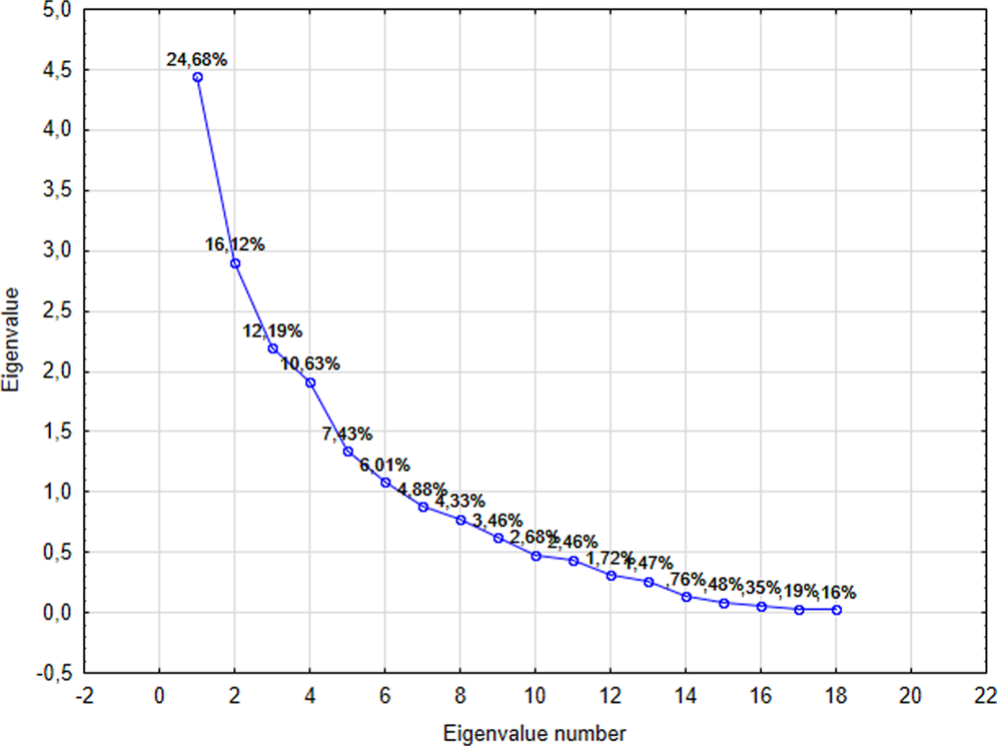
Screen plot with the eigenvalues of the correlation matrix for *A. mellifera* honey samples.

**Table 5:** First Four Principal Component Values and Variances for *A. mellifera* Honey Samples (Atlantic Forest Central Corridor, Southern Bahia, Brazil)

	factor 1	factor 2	factor 3	factor 4
Al	–0.485752	–0.073984	–0.208042	0.377340
Ca	0.333521	0.765597	0.109117	0.789401
Cr	0.740021	0.016662	–0.025465	0.676245
Fe	0.091796	0.039910	–0.571904	0.623337
K	0.807026	0.185320	0.118548	0.752576
Mg	0.169023	0.721399	0.343073	0.823674
Mn	0.598468	0.566622	0.028932	0.700300
Na	0.313537	0.489374	–0.321535	0.559155
Se	–0.675080	0.092952	–0.288682	0.611289
Sr	–0.468226	0.718437	–0.126157	0.766554
Zn	0.409458	0.179318	–0.528353	0.486726
moisture content	–0.118918	–0.112446	0.870258	0.788859
insoluble solid content	0.668886	–0.133525	0.100385	0.572273
total acidity	–0.513914	0.416886	–0.119988	0.459372
diastase activity	–0.174548	0.391289	–0.270474	0.698283
HMF	–0.474974	–0.028874	–0.291742	0.524004
reducing sugar	–0.489622	0.354800	0.320763	0.580985
apparent saccharose	–0.581827	0.390328	0.391748	0.661011
total variance (%)	24.64	16.12	12.19	10.63
cumulative variance (%)	24.64	40.76	52.95	63.58

The results obtained by PCA show a clear tendency for separation between the samples (Figure 2b), forming two main clusters: one composed of wild honeys and the other formed by samples declared as monofloral or predominantly eucalyptus (Eucalyptus spp., Myrtaceae) according to the producers’ indications. It can be observed that orange blossom honey (LMEC, Citrus spp. Rutaceae) and velame honey (VF) were positioned close to the wild honey group, suggesting a possible compositional correlation. This proximity may indicate that these honeys, although marketed as monofloral, share characteristics closer to those of wild multifloral honeys, possibly reflecting an overlap of flowers visited by bees.

**Figure 2:**
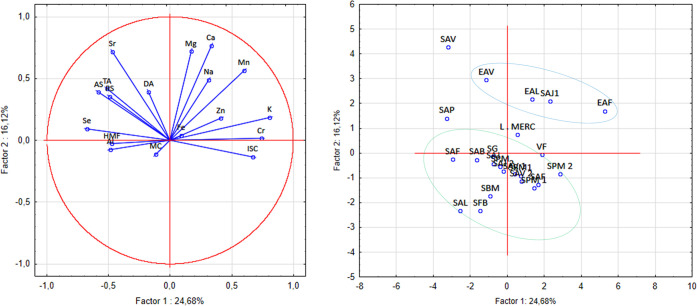
Graph of the first principal component (PC1) versus the second principal component (PC2). (a) PCA score graph and (b) PCA load graph for *A. mellifera* honey samples.

The SAV and SAJ samples, in turn, presented a profile more associated with eucalyptus, which may be related to the proximity of the hives to extensive areas of eucalyptus plantations in southern Bahia, mainly intended for the pulp and paper sector. This regional context strengthens the hypothesis that the agricultural landscape has a direct influence on the chemical and mineral compositions of the honey produced.

The partial overlap observed between certain honeys labeled as monofloral and the group of wild honeys highlights the importance of complementary analyses to confirm botanical authenticity. In this sense, the application of melissopalynology in future studies could provide additional evidence, allowing for the direct identification of pollen grains and the validation of the declared botanical origin. In addition, the integration of palynological data and multielement profiles opens promising prospects for the development of more robust methods of traceability and authentication of honey produced in the region.

Hierarchical cluster analysis (Figure 3) revealed patterns consistent with those observed in PCA. The dendrogram shows the formation of two main groups: one composed predominantly of wild (multifloral) honeys and the other formed mainly of samples declared as monofloral, especially eucalyptus. Some monofloral samples, such as LMEC and VF, clustered closer to the wild honey group, corroborating the scores presented in PCA and suggesting that these honeys may have compositional characteristics typical of multifloral honeys. Similarly, the SAV and SAJ samples appeared close to the eucalyptus samples, reinforcing the high influence of extensive eucalyptus planting in the southern region of Bahia.

**Figure 3:**
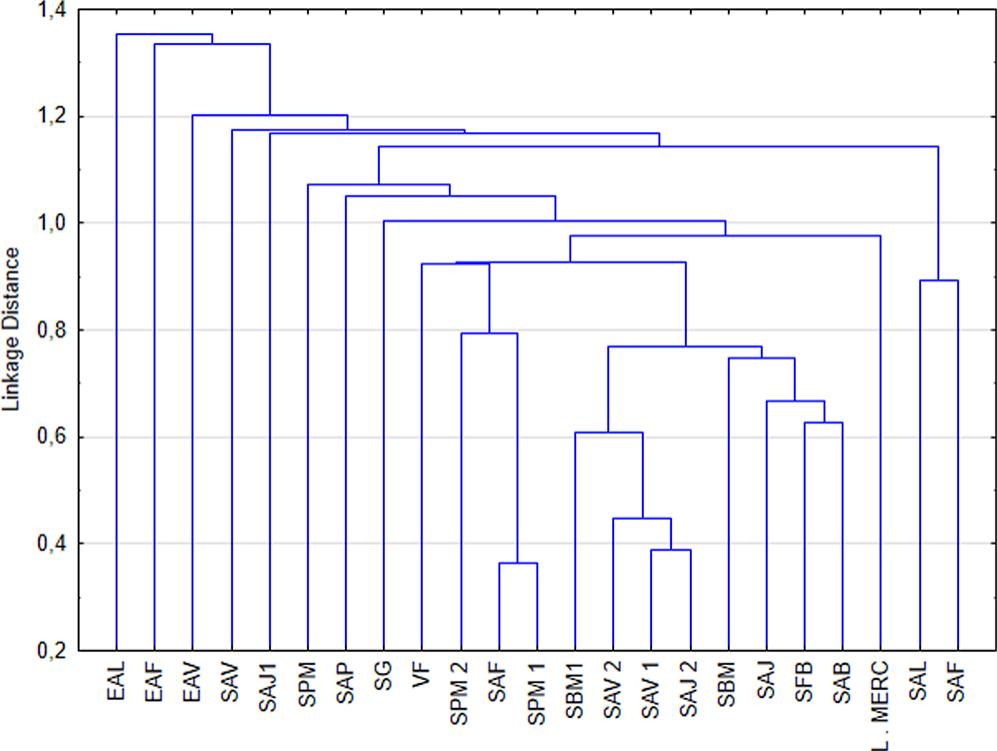
Hierarchical clustering dendrogram for *A. mellifera* honey samples.

## Conclusions

Physical-chemical analyses proved to be an excellent tool for assessing the quality of honey produced and marketed in southern Bahia. These analyses revealed that most samples complied with current Brazilian legislation and international standards, according to the Codex Alimentarius (FAO/WHO), in terms of limits for the physical-chemical parameters.

The relative variation in the concentration of these metals and in the quality of the honey samples is directly linked to their botanical and geographical origins. The analyses confirmed that honey is a food rich in potassium, calcium, magnesium, iron, and sugars, which reinforces its nutritional and medicinal value. Brazilian legislation and the Codex Alimentarius (FAO/WHO) do not provide average values for the metals analyzed.

The PCA technique made it possible to characterize and classify the honey samples according to their indicated blooms. Potassium, chromium, selenium, calcium, magnesium, strontium, and insoluble solids accounted for the greatest variability among the samples. Complementary analyses, including paleopathological studies, may further characterize the profile of honeys and other bee products from the Atlantic Forest of southern Bahia. Future analyses should also include samples of honeys from stingless bees (Apidae: Meliponini), which will make it possible to compare the multielement composition and physicochemical parameters of products from native bees and the Africanized bee, *A. mellifera*.

This study characterizes the chemical profile of honeys from the Central Atlantic Forest Corridor through the integration of multielemental analyses (ICP-OES), physicochemical parameters, and PCA. The approach allowed for the discrimination between mono- and multi-floral honeys, highlighting their potential for authentication and geographic traceability. The results constitute an unprecedented contribution to the evaluation of Brazilian honeys, especially from the Central Atlantic Forest Corridor in Bahia, providing support for the certification of origin and sustainable valorization of bee products from this threatened biome.

## Supplementary Material



## Data Availability

Data are available on request from the corresponding author.

## Notes

LOD, LOQ, and precision for the determination of Al, Ca, Cr, Fe, K, Mg, Mn, Na, Se, Sr, and Zn (PDF)

## References

[1] Dai S., Wellens J., Yang N., Li D., Wang J., Wang L., Yuan S., He Y., Song P., Munger R., Kent M. P., MacFarlane A. J., Mullie P., Duthie S., Little J., Theodoratou E., Li X. (2024). Ultra-processed foods
and human health: An umbrella review and updated
meta-analyses of observational evidence. Clin.
Nutr..

[2] Sanchez-Siles L., Roman S., Fogliano V., Siegrist M. (2022). Naturalness and healthiness
in “ultra-processed foods”: A multidisciplinary perspective
and case study. Trends Food Sci. Technol..

[3] Machado
De-Melo A. A., Almeida-Muradian L. B.
d., Sancho M. T., Pascual-Maté A. (2018). Composition and properties of Apis mellifera honey:
A review. J. Apic. Res..

[4] Jesus M. C., Oliveira D. C., Rodriguez Figueroa L.
E., Brandão H. N., Kamida H. M., Santos F. A. R. (2020). Caracterização botânica
e avaliação do potential antimicrobiano do mel produzido
por Apis mellifera L. Melipona scutellaris Latreille e Tetragonisca
angustula Latreille (Hymenoptera: Apidae) em um fragmento de floresta
ombrófila densa no estado da Bahia, Brasil. Paubrasilia.

[5] Kumar, R. Honey; CRC Press, 2024.

[6] Luca L., Pauliuc D., Oroian M. (2024). Honey microbiota, methods for determining
the microbiological composition and the antimicrobial effect of honey—A
review. Food Chem.:X.

[7] Eshete Y., Belay A. (2023). Botanical Origin, Physicochemical Composition and Antioxidant Content
of Comb, Crushed and Processed Honey Collected from Burie-Ethiopia. Arch. Gynaecol. Women Health.

[8] BRASIL. Ministério da Agricultura e Abastecimento . Instrução Normativa n° 11, de 20 de outubro de 2000. Aprova o Regulamento Técnico de Identidade e Qualidade do Mel. seção 1; Diário Oficial da União: Brasília, DF, 2000, pp 16–18.204

[9] Vîjan L. E., Mazilu I. C., Enache C., Enache S., Topală C. M. (2023). Botanical
Origin Influence on Some Honey Physicochemical Characteristics and
Antioxidant Properties. Foods.

[10] Antunes A. C. N., Gomes V. V., Seraglio S. K. T., Schulz M., Silva B., da Luz C. F. P., de Moraes A. L., Müller M. R. R. P., Gonzaga L. V., Fett R., Costa A. C. O. (2024). Canudo-de-pito
(Escallonia sp.) honey: a comprehensive analysis of quality, composition,
and pollen identification. Eur. Food Res. Technol..

[11] Bana F. C. H., Mocelim M., Ressutte J. B., Cobre A. d. F., Surek M., Hata N. N. Y., Costa V. L. L. d., Cancian M. A. d. Q., Pontarolo R., Macedo F. C., Spinosa W. A. (2025). Honey produced by
apini and meliponini in Brazil: multivariate analysis of physicochemical
parameters, sugar and metabolite profiles. J.
Apic. Res..

[12] Sharin S. N., Abdullah Sani M. S., Kassim N. K., Yuswan M. H., Abd Aziz A., Jaafar M. A., Hashim A. M. (2024). Impact of harvesting seasons on physicochemical
properties and volatile compound profiles of Malaysian stingless bee
honey analysed using chemometrics and support vector machine. Food Chem..

[13] Wisniewski J., Hacke A. C. M., Mazer
Etto R., Boligon A. A., Takeda I., Marques J. A., Pereira R. P. (2024). Evaluation
of the Antioxidant Activity
and Phenolic Composition of Different Monofloral and Polyfloral Brazilian
Honey Extracts. Chem. Biodiversity.

[14] Biswas A., Chaudhari S. R. (2024). Exploring the role of NIR spectroscopy
in quantifying
and verifying honey authenticity: A review. Food Chem..

[15] Elamine Y., Inácio P. M. C., Graça Miguel M.
d., Carlier J. D., Costa M. C., Estevinho L. M., Gomes H. L. (2024). Electrical impedance
spectroscopy for potassium content analysis and botanical origin identification
of honey. Food Chem..

[16] Piana M. L., Cianciabella M., Daniele G. M., Badiani A., Rocculi P., Tappi S., Gatti E., Marcazzan G. L., Magli M., Medoro C., Predieri S. (2023). Influence of the Physical
State of Two Monofloral Honeys on Sensory Properties and Consumer
Satisfaction. Foods.

[17] Živkov
Baloš M., Popov N., Jakšić S., Mihaljev Z. ˇ., Pelić M., Ratajac R., Ljubojević
Pelić D. (2023). Sunflower Honey—Evaluation of Quality and Stability
during Storage. Foods.

[18] Wang H., Li L., Lin X., Bai W., Xiao G., Liu G. (2023). Composition,
functional properties and safety of honey: a review. J. Sci. Food Agric..

[19] Silva B., Antunes A. C. N., Gomes V. V., dos Santos A. C., Schulz M., Seraglio S. K. T., Gonzaga L. V., Fett R., Costa A. C. O. (2024). Brazilian floral honeys: physicochemical, phenolic
compounds, organic acids, and mineral characterization. Eur. Food Res. Technol..

[20] Kamboj R., Nayik G. A., Bera M. B., Nanda V. (2020). Sugar profile
and rheological
behaviour of four different Indian honey varieties. J. Food Sci. Technol..

[21] dos
Santos Silva A., Maciel M. C., Oliveira A. A. F. d., de Oliveira T. F. (2024). Evaluation of the content of macro and trace elements
and the geographic origin of honey in North Brazil through statistical
and machine learning techniques. J. Food Compos.
Anal..

[22] Gregar F., Grepl J., Milde D., Pluháček T. (2024). Direct elemental
analysis of plant oils by inductively coupled plasma mass spectrometry:
Simple sample dilution combined with oxygen introduction into the
plasma. Food Chem..

[23] Luccas F. S., Fernandes E. A. D. N., Mazola Y. T., Bacchi M. A., Sarriés G. A. (2022). Optimization
of sample preparation of Brazilian honeys for TQ-ICP-MS analysis. Talanta.

[24] Ataide
de Oliveira F., Abreu A. T. d., Nascimento N. d. O., Froes R. E. S., Nalini H. A., Antonine Y. (2020). Mineral content in
honey and pollen from native stingless bees Tetragonisca angustula
(Latreille, 1811) in the Iron Quadrangle, Brazil. J. Apic. Res..

[25] Chudzinska M., Debska A., Baralkiewicz D. (2012). Method validation
for determination
of 13 elements in honey samples by ICP-MS. Accredit.
Qual. Assur..

[26] Leme A. B. P., Bianchi S. R., Carneiro R. L., Nogueira A. R. A. (2014). Optimization
of Sample Preparation in the Determination of Minerals and Trace Elements
in Honey by ICP-MS. Food Anal. Methods.

[27] Karabagias I. K., Karabagias V. K., Nayik G. A., Gatzias I., Badeka A. V. (2022). A targeted
chemometric evaluation of the volatile compounds of Quercus ilex honey
in relation to its provenance. LWT.

[28] Karabagias I. K., Badeka A., Kontakos S., Karabournioti S., Kontominas M. G. (2014). Characterisation and classification of Greek pine honeys
according to their geographical origin based on volatiles, physicochemical
parameters and chemometrics. Food Chem..

[29] Sim K. S., Kim H., Hur S. H., Na T. W., Lee J. H., Kim H. J. (2024). Geographical
origin discriminatory analysis of onions: Chemometrics methods applied
to ICP-OES and ICP-MS analysis. Food Res. Int..

[30] Yücel Y., Sultanoglu P. (2013). Characterization of Hatay honeys
according to their
multi-elemental analysis using ICP-OES combined with chemometrics. Food Chem..

[31] Louppis A., Karabagias I., Papastephanou C., Badeka A. (2019). Two-Way Characterization
of Beekeepers’ Honey According to Botanical Origin on the Basis
of Mineral Content Analysis Using ICP-OES Implemented with Multiple
Chemometric Tools. Foods.

[32] Karabagias I. K., Louppis A. P., Karabournioti S., Kontakos S., Papastephanou C., Kontominas M. G. (2017). Characterization
and geographical discrimination of
commercial Citrus spp. honeys produced in different Mediterranean
countries based on minerals, volatile compounds and physicochemical
parameters, using chemometrics. Food Chem..

[33] Liu T., Ming K., Wang W., Qiao N., Qiu S., Yi S., Huang X., Luo L. (2021). Discrimination of honey and syrup-based
adulteration by mineral element chemometrics profiling. Food Chem..

[34] Pinto A. C., Antunes T. J., Santos V. C., Costa C. B. N., Costa J. A. S. (2019). Composição
florística de um fragmento de floresta no Corredor Central
da Mata Atlântica, sul da Bahia, Brasil. Paubrasilia.

[35] Rezende C. L., Scarano F. R., Assad E. D., Joly C. A., Metzger J.-P., Strassburg B. B. N., Tabarelli M., Fonseca G. A., Mittermeier R. A. (2018). From hotspot
to hopespot: An opportunity for the Brazilian Atlantic Forest. Perspect. Ecol. Conserv..

[36] Instituto Adolfo Lutz . Capítulo 7—Açúcares e produtos correlatos. In Métodos Físico-Químicos Para Análise de Alimentos, 4th ed.; Zenebon, O. ; Pascuet, N. S. ; Tiglea, P. , Eds.; Instituto Adolfo Lutz: São Paulo, 2008; pp 321–343.

[37] INMETRO, Instituto Nacional de Metrologia, Q. e T . Orientação Sobre Validação de Métodos Analíticos, 2020.

[38] Barreiros J., Cepeda A., Franco C., Nebot C., Vázquez B. (2024). Analysis of
minerals in honey and their nutritional implications. J. Food Compos. Anal..

[39] Flora e Funga do Brasil . Jardim Botânico do Rio de Janeiro. http://floradobrasil.jbrj.gov.br (accessed March 25, 2025).

[40] de
Souza R. R., de Abreu V. H. R., de Novais J. S. (2019). Melissopalynology
in Brazil: A map of pollen types and published productions between
2005 and 2017. Palynology.

[41] Bandeira M. S. F., Novais J. S. (2021). Brazilian peppertree,
eucalyptus, and velame honeys:
Does palynology confirm the predominant flower sources indicated by
beekeepers?. An. Acad. Bras. Ciênc..

[42] da
Silva P. M., Gauche C., Gonzaga L. V., Costa A. C. O., Fett R. (2016). Honey: Chemical composition, stability and authenticity. Food Chem..

[43] Guiné R. P. F., Florença S. G., Correia P. M. R., Anjos O., Coelho C., Costa C. A. (2022). Honey Bee
(Apis mellifera L.) Broods:
Composition, Technology and Gastronomic Applicability. Foods.

[44] Abeslami A., El Farissi H., Cacciola F., El Bachiri A., Sindic M., Fauconnier M.-L., Bruneau E., Talhaoui A. (2025). Unveiling
the Mineral and Sugar Richness of Moroccan Honeys: A Study of Botanical
Origins and Quality Indicators. Molecules.

[45] Bora F. D., Andrecan A. F., Călugăr A., Bunea C. I., Popescu M., Petrescu-Mag I. V., Bunea A. (2024). Comprehensive Elemental
Profiling of Romanian Honey: Exploring Regional Variance, Honey Types,
and Analyzed Metals for Sustainable Apicultural and Environmental
Practices. Foods.

[46] Biluca F. C., de Gois J. S., Schulz M., Braghini F., Gonzaga L. V., Maltez H. F., Rodrigues E., Vitali L., Micke G. A., Borges D. L. G., Costa A. C. O., Fett R. (2017). Phenolic compounds,
antioxidant capacity, and bioaccessibility of minerals of stingless
bee honey (Meliponinae). J. Food Compos. Anal..

[47] Moniruzzaman M., Chowdhury M. A. Z., Rahman M. A., Sulaiman S. A., Gan S. H. (2014). Determination
of Mineral, Trace Element, and Pesticide Levels in Honey Samples Originating
from Different Regions of Malaysia Compared to Manuka Honey. Biomed. Res. Int..

[48] Nicolson S. W. (2022). Sweet solutions:
nectar chemistry and quality. Philos. Trans.
R. Soc., B.

[49] Nicolson S. W., Worswick W. P. V. (1990). Sodium and potassium concentrations
in floral nectars
in relation to foraging by honey bees. Afr.
Zool..

[50] Buffon S. B., Zucoloto M., Passos O. S., Barbosa D. H. S. G., Altoé M. S., Morais A. L. d. (2021). Initial production
and fruit quality
of fifty-seven sweet orange varieties on four rootstocks in southern
state of Bahia. Rev. Bras. Frutic..

[51] Oliveira V. d. S., Zucoloto M., Ferreira L. d. S., Barbosa D. H. S. G., Soares Filho W., Passos O. S. (2024). Productive performance of sweet orange
trees on different rootstocks, in the Extreme South of the State of
Bahia. Rev. Bras. Frutic..

[52] Souza E. C. A., Cavalcante dos Santos Campos D., da Costa L. A. M. A., de Mello Pereira F., Menezes C., Flach A. (2025). Effect of
pasteurization,
dehumidification, refrigeration and maturation on the composition,
quality and acceptability of Scaptotrigona depilis honey. J. Apic. Res..

[53] Food and Agriculture Organization of the United Nations . FAOSTAT—Food Balances https://www.fao.org/faostat/en/#data/FBS. (accessed July 10, 2025).

[54] Florentino A. P., Lima C. d. V., Morandini G. G., Silva G. C., Morelli L. M., Battistin L., Pivato M. E. B., Silva M. S. F., Vieira M. F., Canute M. d. S., Paulino M. S. d. M., Araujo P. C., Cravo B. R., Silvestre V. R., Neckel L. G. d. S., Martins O. A., Sampaio A. N. d. C. E., Possebon F. S., Pereira J. G. (2023). High frequency of fraud in honey
without sanitary inspection illegally sold in São Paulo State. Food Sci. Technol..

[55] FAO, OMS . Codex Standard for Honey (CXS 12-1981), 2022.

[56] MERCOSUL . Regulamento Técnico MERCOSUL “Identidade e Qualidade do Mel”. GMC/RES. N° 89/99, 1999.

[57] Evans D. E., Fox G. P. (2017). Comparison of Diastase
Power Enzyme Release and Persistence
during Modified Institute of Brewing 65°C and Congress Programmed
Mashes. J. Am. Soc. Brew. Chem..

[58] Makhloufi C., Taïbi K., Ait Abderrahim L. (2020). Characterization of Invertase and
Diastase Activities, 5-Hydroxymethylfurfural Content and Hydrogen
Peroxide Production of Some Algerian Honeys. Iran. J. Sci. Technol., Trans. A: Sci..

[59] Schiassi M. C. E. V., de Souza V. R., Lago A. M. T., Carvalho G. R., Curi P. N., Guimarães A. S., Queiroz F. (2021). Quality of honeys from different
botanical origins. J. Food Sci. Technol..

[60] Wang H., Geppert H., Fischer T., Wieprecht W., Möller D. (2015). Determination of Sucrose in Honey
with Derivatization/Solid-Phase
Microextraction and Gas-Chromatography/Mass Spectrometry. J. Chromatogr. Sci..

[61] Waworuntu J. (2024). Sugar Content
Composition of Various Types of Honey Produced by Apis Mellifera L.:
A Review. JPPIPA.

