# Supplementary material for: Compositional Assessment of Honeys from the Central Atlantic Forest: Multielement and Physicochemical Characterization
Source: ACS Omega. 2025 Oct 12;10(41):48700–10. doi: 10.1021/acsomega.5c06565 (PMC12547541; doi:10.1021/acsomega.5c06565)
Supplement: Supplementary file 1 [file ao5c06565_si_001.pdf]

Table S1 - Detection limits (LOD), quantification limits (LOQ), and precision (expressed as relative standard deviation) for the determination of Al, Ca, Cr, Fe, K, Mg, Mn, Na, Se, Sr, and Zn.

	**LOD (mg Kg^{-1})**	**LOQ (mg Kg^{-1})**
Al	0.0037	0.0123
Ca	0.2192	0.7307
Cr	0.0150	0.0501
Fe	0.1309	0.4365
K	0.4177	1.3924
Mg	0.0851	0.2836
Mn	0.0165	0.0549
Na	0.5120	1.7067
Se	0.0597	0.1990
Sr	0.0421	0.1404
Zn	0.1575	0.5251
